# Dynamics of GnRH Neuron Ionotropic GABA and Glutamate Synaptic Receptors Are Unchanged during Estrogen Positive and Negative Feedback in Female Mice

**DOI:** 10.1523/ENEURO.0259-17.2017

**Published:** 2017-11-06

**Authors:** Xinhuai Liu, Robert Porteous, Allan E. Herbison

**Affiliations:** Centre for Neuroendocrinology and Department of Physiology, University of Otago School of Biomedical Medical Sciences, Dunedin 9054, New Zealand

**Keywords:** electrophysiology, estradiol, GABA, glutamate, GnRH

## Abstract

Inputs from GABAergic and glutamatergic neurons are suspected to play an important role in regulating the activity of the gonadotropin-releasing hormone (GnRH) neurons. The GnRH neurons exhibit marked plasticity to control the ovarian cycle with circulating estradiol concentrations having profound “feedback” effects on their activity. This includes “negative feedback” responsible for suppressing GnRH neuron activity and “positive feedback” that occurs at mid-cycle to activate the GnRH neurons to generate the preovulatory luteinizing hormone surge. In the present study, we employed brain slice electrophysiology to question whether synaptic ionotropic GABA and glutamate receptor signaling at the GnRH neuron changed at times of negative and positive feedback. We used a well characterized estradiol (E)–treated ovariectomized (OVX) mouse model to replicate negative and positive feedback. Miniature and spontaneous postsynaptic currents (mPSCs and sPSCs) attributable to GABA_A_ and glutamatergic receptor signaling were recorded from GnRH neurons obtained from intact diestrous, OVX, OVX + E (negative feedback), and OVX + E+E (positive feedback) female mice. Approximately 90% of GnRH neurons exhibited spontaneous GABA_A_-mPSCs in all groups but no significant differences in the frequency or kinetics of mPSCs were found at the times of negative or positive feedback. Approximately 50% of GnRH neurons exhibited spontaneous glutamate mPSCs but again no differences were detected. The same was true for spontaneous PSCs in all cases. These observations indicate that the kinetics of ionotropic GABA and glutamate receptor synaptic transmission to GnRH neurons remain stable across the different estrogen feedback states.

## Significance Statement

The gonadotropin-releasing hormone (GnRH) neurons are the key output cells controlling fertility in all mammals. Glutamatergic and GABAergic inputs are hypothesized to play a key role in mediating the so-called “negative” and “positive” feedback actions of circulating estradiol on GnRH neuron firing. These feedback actions are critical for the GnRH neurons to control the fluctuating levels of reproductive hormones that occur across the ovarian cycle. We show here that the dynamics of synaptic ionotropic GABA and glutamate receptors on GnRH neurons do not change in relation to estradiol negative and positive feedback. This indicates that changes in presynaptic GABA/glutamate release may be more important that postsynaptic mechanisms in the control of GnRH neuron firing across the ovarian cycle.

## Introduction

Inputs from glutamatergic and GABAergic neurons are thought to play a major role in defining the electrical behavior of the gonadotropin-releasing hormone (GnRH) neurons that control fertility (Jennes et al., 2002; Moenter et al., 2009; Iremonger et al., 2010; Herbison and Moenter, 2011). Essentially all GnRH neurons express functional AMPA, GABA_A_, and GABA_B_ receptors with sub-populations having NMDA and kainate receptors (Iremonger et al., 2010; Herbison and Moenter, 2011). Whereas the activation of GABA_B_ receptors inhibits the electrical excitability of GnRH neurons (Zhang et al., 2009; Liu and Herbison, 2011), the activation of ionotropic GABAergic and glutamatergic receptors is excitatory (Yin et al., 2008; Iremonger et al., 2010; Herbison and Moenter, 2011). While it is clear that amino acid transmitters can have profound effects on the electrical excitability of GnRH neurons, their physiologic roles remain uncertain.

Many studies have examined the effects of manipulating hypothalamic glutamate and GABA_A_ receptor occupancy on the secretion of gonadotropins *in vivo*. These have suggested key roles for amino acid transmitters in the onset of puberty (Terasawa and Fernandez, 2001; Clarkson and Herbison, 2006), generation of pulsatile and surge modes of luteinizing hormone (LH) secretion (Brann and Mahesh, 1997; Jackson and Kuehl, 2002; Mitsushima et al., 2003; Herbison, 2015) and seasonal transitions in fertility (Scott and Clarke, 1993; Jansen et al., 2003). However, as all neurons express ionotropic GABA and glutamate receptors, it has been difficult to assess the functional impact of amino acid signaling directly at the GnRH neuron. The same difficulty exists for *in vivo* microinfusion investigations that have attempted to modulate GABA_A_ receptor occupancy within the immediate vicinity of the GnRH neuron cell bodies (Herbison et al., 1991; Jarry et al., 1991). Furthermore, investigations using mice with GnRH neuron-selective deletion or knockdown of GABA_A_ and NMDA receptors have also failed to offer any clear insight into the physiologic roles of direct amino acid signaling at the GnRH neuron (Shimshek et al., 2006; Lee et al., 2010).

An alternative approach to understanding the significance of GABA and glutamate signaling at the GnRH neuron has been to use whole-cell patch clamp electrophysiology in acute brain slices. This has enabled the dynamics of GABA and glutamate receptors expressed by GnRH neurons to be assayed over a range of different experimental conditions (Moenter, 2010; Herbison and Moenter, 2011). Of particular importance, studies by the Moenter laboratory have revealed robust changes in the frequency and amplitude of amino acid postsynaptic currents (PSCs) exhibited by GnRH neurons during estrogen negative and positive feedback. In terms of negative feedback, the frequency of both GABA_A_ and AMPA receptor PSCs, and amplitude of AMPA PSCs, were reduced in ovariectomized (OVX) plus estrogen (OVX + E) mice compared with OVX animals (Christian and Moenter, 2007; Christian et al., 2009). At the time of positive feedback just before or during the LH surge, both the frequency and amplitude of GABA_A_ PSCs were enhanced while no change was observed for AMPA PSCs (Christian and Moenter, 2007; Christian et al., 2009). Compatible with the substantial loss of neural inputs to cells in brain slices, these changes in PSC frequency arose from alterations in spontaneous vesicle release rather than action potential-dependent release of amino acids. Nevertheless, these findings indicated that synaptic GABA transmission at the GnRH neuron was changing at times of both positive and negative feedback, whereas AMPA receptor dynamics rearranged only during negative feedback. Although correlative, these results suggested that GABA and glutamate inputs to GnRH neurons were involved in transitioning GnRH neuron behavior across the estrous cycle (Moenter et al., 2009). This is an important concept given that the GnRH neurons themselves do not express estrogen receptor-α (ESR1), the receptor mediating estrogen negative and positive feedback (Herbison, 2015; Levine, 2015).

The limitation of prior GABA and glutamate PSC studies on GnRH neurons is that they were all undertaken in an unusual mouse model in which mice fluctuate between negative and positive feedback on a daily basis in response to constant high levels of estradiol (Christian et al., 2005). This is unlike the physiologic situation where serum estradiol levels increase gradually over 3 d to generate positive feedback, with negative feedback being present at all other times (Herbison, 2015; Levine, 2015). As such, we were interested in re-assessing GABA and glutamate PSCs in GnRH neurons in mouse models that more closely follow the natural estrous cycle. Developed originally by Bronson (Bronson, 1981), these models involve ovariectomy and immediate insertion of an estradiol-filled SILASTIC capsule to provide low levels of serum estradiol that generate only negative feedback. After 5 d, these negative feedback OVX + E mice can then be given a supplemental injection of estradiol benzoate (OVX + E+E) to mimic the diestrous/proestrous rise in estradiol that generates positive feedback and the LH surge 2 d later. Using these mouse models, and intact diestrous mice to compare with OVX + E animals, we have now re-examined whether glutamate and GABA_A_ receptor PSCs are altered in GnRH neurons during negative and positive feedback.

## Materials and Methods

### Mouse models

Adult female (131 ± 5 d of age) C57BL/6 GnRH-GFP mice (Spergel et al., 1999) were housed under a 12/12 h light/dark cycle (lights on 6 A.M., off at 6 P.M.) with ad libitum access to food and water in groups of two or three animals per cage. Vaginal cytology of intact females was examined daily at 10 A.M. to determine the diestrous stage of the estrous cycle for mice used in the intact control group. Other mice were anaesthetised with isoflurane anesthesia and OVX 7-10 d before being used in electrophysiological experiments. “OVX” mice received no estradiol treatments. “OVX + E mice” were implanted with subcutaneous SILASTIC implants containing 17β-estradiol (1 µg/20 g body weight; Bronson, 1981) at the time of ovariectomy and used for electrophysiology experiments 7-10 d (mode = 8) later. At this time OVX + E mice exhibit negative feedback with pulsatile LH secretion slowed to the same frequency as intact diestrous mice (Czieselsky et al., 2016). OVX, OVX + E, and diestrous mice were killed at 1:00-1:30 P.M. and brain slices recorded from 2:30-5:00 P.M. A further group of OVX + E mice were given a subcutaneous injection of estradiol benzoate (1 µg/100 µl) at 9 A.M. 6 d after SILASTIC capsule implantation (OVX + E+E; Bronson, 1981) and used for electrophysiology experiments the following day. These OVX + E+E mice were killed at 4:00-4:30 P.M. and brain slices recorded at “surge onset” from 5:00-7:30 P.M. OVX + E+E mice exhibit an LH surge that starts at ∼5:30 P.M. (Czieselsky et al., 2016). All animal procedures were performed in accordance with the University of Otago animal care committee's regulations.

### Brain slice preparation and electrophysiology

The dissected brain was glued to a round aluminum platform and then submerged in the cooled (∼2°C) artificial CSF (aCSF) containing high (6 mM) MgCl_2_ and low (0.5 mM) CaCl_2_ and equilibrated with 95% O_2_, 5% CO_2_. The 250-μm-thick sagittal brain slices were cut using a vibratome (Leica VT1000S). Sagittal slices were used based on prior work showing sagittal slices to exhibit the most dynamic PSC changes in estrogen-treated OVX mice (Christian and Moenter, 2007). Brain slices were incubated for at least 1h in equilibrated (95% O_2_, 5% CO_2_) aCSF, containing 118 mM NaCl, 3 mM KCl, 2.5 mM CaCl_2_, 1.2 mM MgCl_2_, 11 mM D-glucose, 10 mM HEPES, and 25 mM NaHCO_3_ with pH 7.3 at 30°C, before being transferred to a submerged recording chamber where they were perfused with aCSF at 2-3 ml/min maintained at 32 ± 1°C. Whole-cell recordings of GnRH neurons were undertaken using a fixed-stage upright fluorescence microscope (BX51WI; Olympus) with GFP-tagged GnRH neurons identified briefly (<10 s) using fluorescence and then patched under Nomarski differential interference contrast optics (40× water-immersion objective). Patch pipettes were pulled from glass capillaries (inner diameter, 1.17 mm; outer diameter, 1.5 mm) with a microelectrode puller (Sutter Instruments) and had 4–6 MOhm resistance when filled with the pipette solution composed of 130 mM K-gluconate (glutamate PSCs) or 130 mM KCl (GABA_A_ PSCs), 5 mM NaCl, 0.22 mM CaCl_2_, 10 mM HEPES, 2 mM BAPTA, 2 mM MgATP, 2 mM Na_2_ATP, 0.2 mM Na_2_GTP, and 10 mM phosphocreatine-Tris (pH 7.35 adjusted by KOH, ∼290 mOsmol).

Signals (voltage and current) were amplified with a Multiclamp 700B amplifier (CV7B; Molecular Devices) and sampled on-line with the use of a Digidata 1440A interface (Molecular Devices) connected to a personal computer. Signals were filtered with Bessel filter of Multiclamp 700B (at 3 KHz for current or 10 kHz for voltage) before being digitized at a rate of 10 kHz. Acquisition and subsequent analysis of the acquired data were performed with the Clampex 10 suite of software (Molecular Devices) and Origin Pro 7.5 (OriginLab). Resting membrane potential was determined in current clamp without any holding current and liquid junction potentials of ∼12 mV uncorrected for the gluconate-based solution. The input resistance was determined by pClampex 10 membrane test while holding the cell at -70 mV. During experiments, the access resistance (Ra = 12 ± 0.3 MΩ, *N* = 138) was checked regularly.

After attaining a stable whole-cell voltage-clamp recording held at -70 mV, the aCSF was switched to one containing the appropriate amino acid receptor antagonists (see below), and 2 min later, spontaneous PSCs (sPSCs) were recorded for 3-4 min. The perfusion medium was then changed to one containing tetrodotoxin (TTX), in addition to the amino acid receptor antagonists, and 2 min later miniature PSCs (mPSCs) recorded for 3-4 min. At the conclusion of the recording, the brain slice was removed and a new slice used for the next cell. Two to four cells were obtained from each animal. If a healthy cell did not exhibit PSCs, its PSC mean frequency was considered to be zero with no parameters being measured. A cell was considered to be healthy if its input resistance was >450 MΩ, series resistance ≤23 MΩ (without change during the data collection), membrane capacitance ≥14 pF, resting membrane potential below -47 mV and action potential amplitude >80 mV.

### Drugs

Stock solutions of D-2-amino-5-phosphonovaleric acid (AP5), GABAzine, 6-cyano-7-nitroquinoxaline-2,3-dione (CNQX; Sigma Ltd), and TTX (Alomone Labs) were prepared in double distilled H_2_O at 10^3^ times final concentration. All stock solutions were stored at -20°C. All drugs were applied in the perfusion solution with final concentration as indicated. The mixture of CNQX (10 μM) and AP5 (50 μM) was used to block all glutamatergic ionotropic receptor-mediated synaptic transmission. GABAzine (5 μM) was used to block all GABAergic ionotropic receptor-mediated synaptic transmission. TTX (0.5 μM) was used to block all action potential-dependent transmission.

### Analysis

PSCs were detected by the MiniAnalysis program (Synptosoft, NJ07024) and confirmed by eye with detection errors corrected manually. All PSCs occurring over two 3-min periods were analyzed for each cell with a maximum of 300 PSCs/period being analyzed for any one cell. Analysis parameters included mean frequency (number of PSCs divided by 180 s or, for cells displaying many PSCs, the number of seconds during which 300 PSCs occurred), average inter-PSCs interval, PSC amplitude, rise time between 10% and 90% amplitude of PSCs, decay time between 10% and 90% amplitude of PSCs, and half width of PSCs. GABAergic PSCs, termed GPSCs, were obtained in the presence of AP5 (50 μM) + CNQX (10 μM) in the perfusion solution. In separate experiments, glutamatergic PSCs, termed excitatory PSCs (EPSCs) were obtained in the presence of GABAzine (5 μM) in the perfusion solution. mPSCs were assessed by adding TTX (0.5 μM) to the aCSF and analyzed in the same manner for sPSCs detailed above. Signals were passed through a low-pass filter (1 KHz) and average PSCs and mPSCs traces obtained for each cell by aligning all events on their rising phase and these were then averaged across each experimental group.

### Statistical analysis

The percentages of GnRH neurons exhibiting PSCs were compared using the χ^2^ test (among four groups) or Fisher’s exact test (between two groups). Analysis of the mean frequency, inter-PSCs interval, amplitude, rise time, decay time, and half-width of PSCs was undertaken with Kruskal-Wallis nonparametric ANOVA. To compare cumulative frequency of the inter-PSC intervals or amplitude of PSCs, mean, mean + 2 SD, and mean - 2 SD were pooled before applying Kruskal-Wallis nonparametric ANOVA. In all analyses, differences were considered statistically significant at *p* < 0.05.

## Results

### Miniature GABA PSCs (mGPSCs)

**Figure 1.**
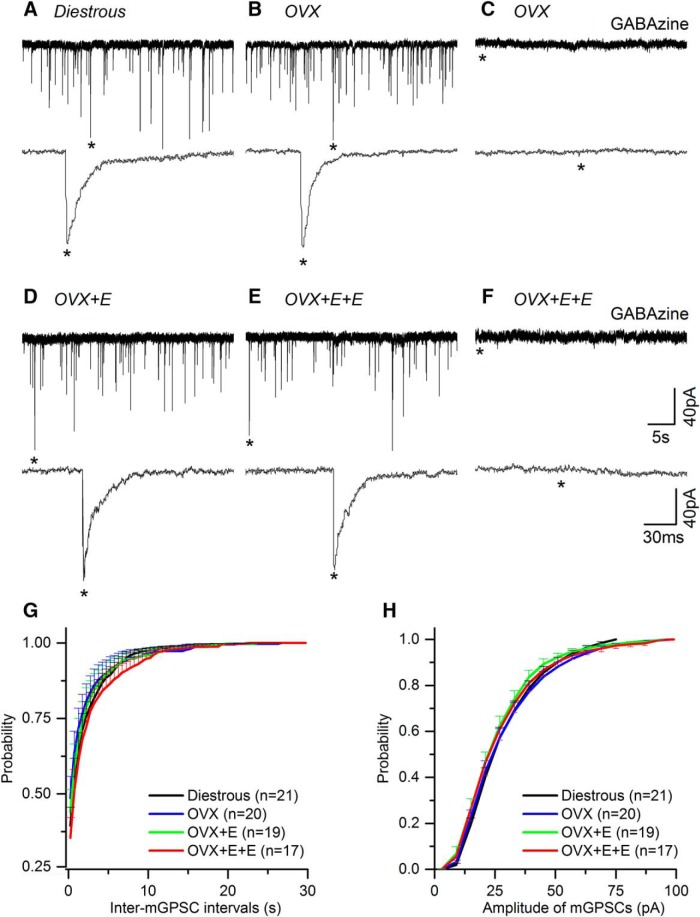
Miniature GABA_A_ receptor PSCs (mGPSCs) recorded from GnRH neurons do not change in the different mouse models of estrogen negative and positive feedback. ***A****-****F***, Representative examples of mGPSC current recordings in the presence of AP5 (50 μM), CNQX (10 μM), and TTX (0.5 μM) taken from diestrous, OVX, OVX + E, and OVX + E+E mice. Underneath each trace an enlarged time scale of one mGPSC (*) is shown. ***C***, ***F***, mGPSCs are abolished by GABAzine (5 μM). ***G***, ***H***, Cumulative plots of the average inter-mGPSC interval (***G***) and amplitude (***H***) in the different experimental groups (for clarity only positive SEM is shown). *N* = 7 for each group. Cell numbers are shown in parenthesis. Numbers of PSCs analyzed is given in Table 1. No statistically significant differences were detected for any parameter between groups.

In total, seven GnRH-GFP mice were used in each of the four experimental groups, yielding mGPSC recordings from 24 (diestrous), 21 (OVX), 20 (OVX + E), and 22 (OVX + E+E) GnRH neurons. Recorded cells were located predominantly within the rostral preoptic area with 4% (six of 138 cells) located in the lateral aspects of the anterior hypothalamus. Recordings undertaken in AP5, CNQX, and TTX, revealed typical mGPSCs (Fig. 1*A*,*B*,*D*,*E*) that were abolished completely by adding 5 μM GABAzine to the aCSF (Fig. 1*C*,*F*).

**Figure 2.**
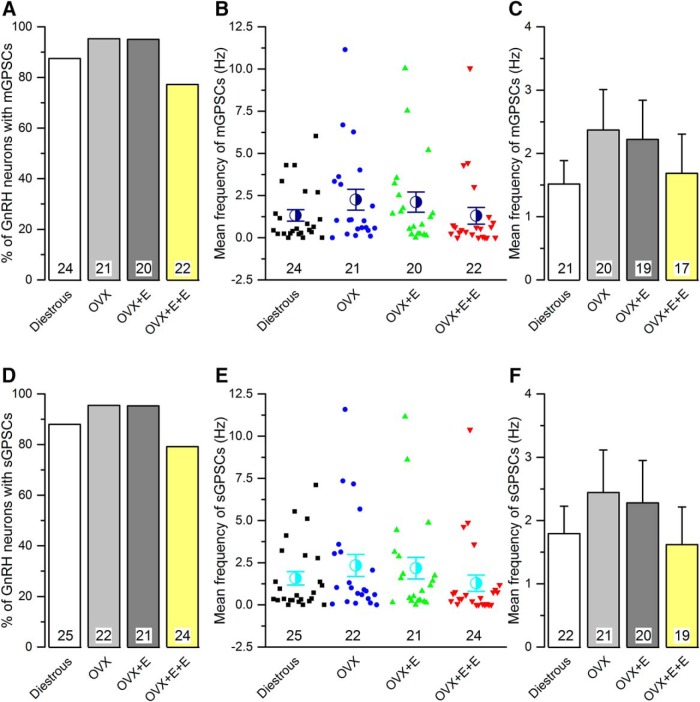
The frequency of miniature (mGPSC) and spontaneous (sGPSC) GABA_A_ receptor PSCs recorded from GnRH neurons do not change in the different mouse models of estrogen negative and positive feedback. ***A***, ***D***, Histograms showing the percentage of GnRH neurons exhibiting mGPSCs (***A***) or sGPSCs (***D***) in each group. ***B***, ***E***, Scatter plots showing the individual and mean (±SEM) frequency of mGPSCs (***B***) or sGPSCs (***E***) in all GnRH neurons of the four experimental groups. ***C***, ***F***, Histograms showing the mean frequency of mGPSCs (***C***) or sGPSCs (***F***) from only those cells exhibiting mGPSCs (***C***) or GPSCs (***F***) in each animal group. Numbers at the base show the cell number. The animal number is seven for each group. No statistically significant differences were detected for any parameter between the different groups.

No differences were detected in the occurrence, frequency, or dynamics of mGPSCs between diestrous and OVX mice (Figs. 1, 2; Table 1). Approximately 90% of all GnRH neurons exhibited mGPSCs in both groups (Fig. 2*A*) and regardless of whether cumulative probability (Fig. 1*G*,*H*) or group mean events (Fig. 2*A-C*) were analyzed, the inter mGPSC interval and mGPSC amplitude were not different. Similarly, the mean frequency of mGPSCs over the 3-min recording period was not different when all cells were tested (Fig. 2*B*) or when only cells exhibiting mGPSCs were analyzed (Fig. 2*C*). Rise time, decay time and half-width of GPSCs were also not different (Table 1).

**Table 1.** Summary of GPSCs and mGPSCs in different experimental groups

		*n* (cells)	PSCs analyzed	Inter-PSCs intervals (s)	amplitude (pA)	10-90% rise time (ms)	10-90% Decay time (ms)	Half width (ms)
Diestrus (*N* = 7)	GPSCs mGPSCs	22 21	3814 3453	1.92 ± 0.37 2.04 ± 0.35	33.83 ± 2.403 3.46 ± 2.10	1.35 ± 0.07 1.38 ± 0.07	10.76 ± 0.46 11.15 ± 0.49	6.28 ± 0.37 6.34 ± 0.39
OVX (*N* = 7)	GPSCs mGPSCs	21 20	3219 3184	3.09 ± 1.20 1.97 ± 0.64	31.71 ± 1.87 33.17 ± 1.66	1.55 ± 0.10 1.54 ± 0.10	10.27 ± 0.59 10.30 ± 0.64	6.10 ± 0.40 6.39 ± 0.41
OVX+E (*N* = 7)	GPSCs mGPSCs	20 19	2816 2571	2.18 ± 0.55 1.98 ± 0.51	30.94 ± 1.81 30.51 ± 1.73	1.60 ± 0.08 1.57 ± 0.08	11.15 ± 0.80 10.98 ± 0.84	6.67 ± 0.47 6.72 ± 0.62
OVX+E+E (*N* = 7)	GPSCs mGPSCs	19 17	3636 3535	5.00 ± 1.84 3.21 ± 1.11	31.21 ± 2.32 31.55 ± 2.37	1.50 ± 0.11 1.41 ± 0.10	10.43 ± 0.54 10.41 ± 0.57	6.00 ± 0.37 6.07 ± 0.40

No statistically significant differences were detected for any parameter between the groups.

The same result was found when comparing OVX with OVX + E and OVX + E+E recordings with no statistically significant differences being detected in terms of the numbers of GnRH neurons exhibiting mGPSCs (Fig. 2*A*) or the frequency and dynamics of mGPSCs (Figs. 1, 2; Table 1).

### Spontaneous GPSCs (sGPSCs)

**Figure 3.**
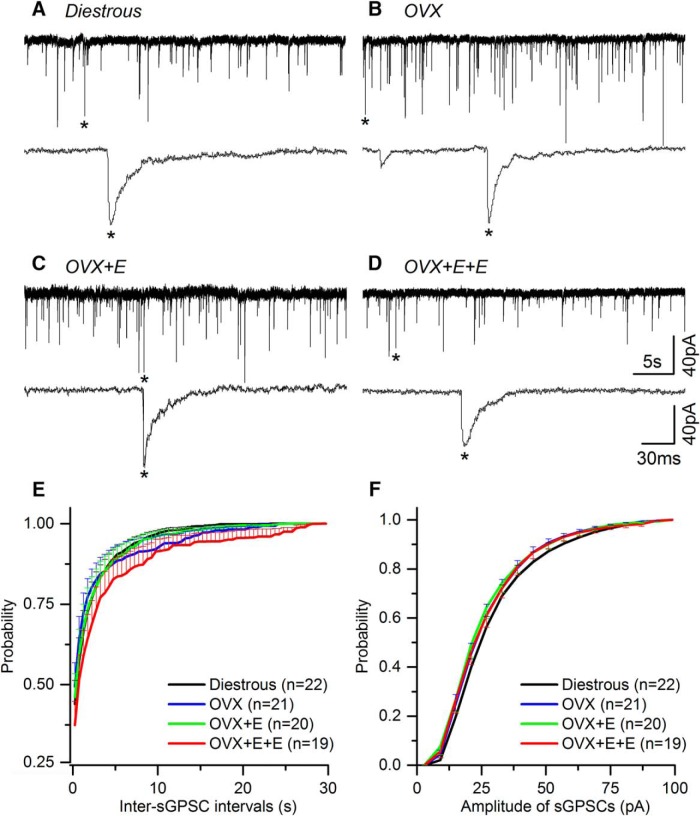
Spontaneous GABA_A_ receptor PSCs (sGPSCs) recorded from GnRH neurons do not change in the different mouse models of estrogen negative and positive feedback. ***A****-****D***, Representative examples of sGPSC current recordings in the presence of AP5 (50 μM) and CNQX (10 μM) taken from diestrous, OVX, OVX + E, and OVX + E+E mice. Underneath each trace, an enlarged time scale of one GPSC (*) is shown. ***E***, ***F***, Cumulative plots of the average inter-sGPSC interval (***G***) and amplitude (***H***) in the different experimental groups (for clarity only positive SEM is shown). *N* = 7 for each group. Cell numbers are shown in parenthesis. Numbers of PSCs analyzed is given in Table 1. No statistically significant differences were detected for any parameter between groups.

sGPSCs were recorded from 25 (diestrous), 22 (OVX), 21 (OVX + E), and 24 (OVX + E+E) GnRH neurons, 94% of which yielded the mGPSC results above. Recordings were undertaken in AP5 and CNQX and revealed typical sGPSCs (Fig. 3*A*-*D*).

As was found for the mGPSCs, no significant differences were detected for any sGPSC parameter between any of the experimental groups (Figs. 2*D-F*, 3*A-F* ; Table 1).

**Figure 4.**
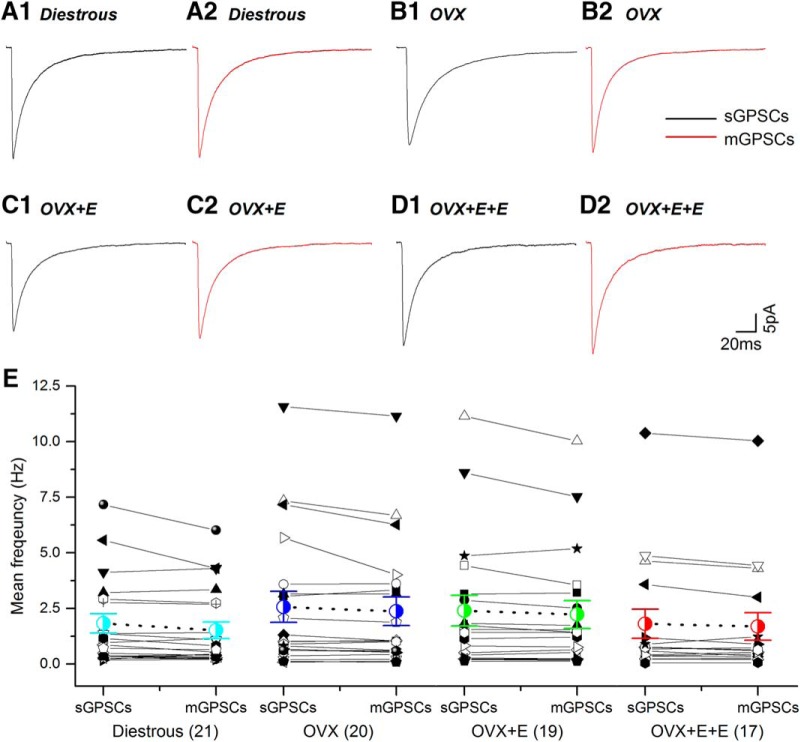
The kinetics and mean frequency of spontaneous (sGPSCs) and miniature (mGPSCs) PSCs are not statistically different to one another and do not change during negative and positive feedback. ***A1-D2***, Average waveforms of sGPSCs (black) and mGPSCs (red) are depicted for each experimental group. ***E***, sGPSC and mGPSC values for individual cells (various black symbols) are plotted with group mean (±SEM) indicated in colored symbol with dashed line. Number of individual cells in each group are given in brackets. No statistically significant differences were detected for any parameter between the different groups.

No significant differences were detected between mGPSCs and sGPSCs (Fig. 4; Table 1).

### Miniature EPSCs (mEPSCs)

**Figure 5.**
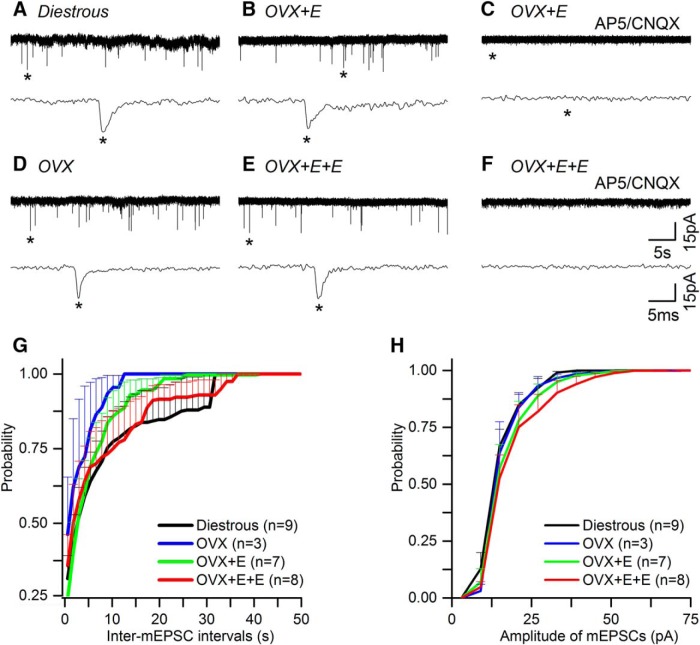
Miniature excitatory glutamate receptor PSCs (mEPSCs) recorded from GnRH neurons do not change in the different mouse models of estrogen negative and positive feedback. ***A****-****F***, Representative examples of mEPSCs current recordings in the presence of GABAzine (5 μM) and TTX (0.5 μM) taken from diestrous, OVX, OVX + E, and OVX + E+E mice. Underneath each trace an enlarged time scale of one mEPSC (*) is shown. ***C***, ***F***, mEPSCs are abolished by AP5 (50 μM) + CNQX (10 μM). ***G***, ***H***, Cumulative plots of the average inter-mEPSC interval (***G***) and amplitude (***H***) in the different experimental groups (for clarity only positive SEM is shown). *N* = 5 for each group. Cell numbers are shown in parenthesis. Numbers of PSCs analyzed is given in Table 2. No statistically significant differences were detected for any parameter between groups.

In total, five GnRH-GFP mice were used for each of the four experimental groups, yielding mEPSC recordings from 16 (diestrous), 13 (OVX), 15 (OVX + E), and 16 (OVX + E+E) GnRH neurons. Recordings undertaken in the presence of GABAzine and TTX revealed mEPSCs typical of GnRH neurons (Fig. 5*A*,*B*,*D*,*E*) that were abolished by adding AP5 (50 μM) + CNQX (10 μM) to the aCSF (Fig. 5*C*,*F*). The mean half-width of mEPSCs is ∼1 ms (Table 2) compared with ∼6 ms for mGPSCs (Table 1), suggesting that the great majority of mEPSCs recorded under the present conditions represent AMPA PSCs (Liu et al., 2011).

**Table 2.** Summary of EPSCs and mEPSCs in different experimental groups

		*N* (cells)	PSCs analyzed	Inter-PSCs intervals (s)	amplitude (pA)	10-90% rise time (ms)	10-90% Decay time (ms)	Half width (ms)
Diestrus (*N* = 5)	EPSCs mEPSCs	11 9	899 575	12.56 ± 7.19 14.39 ± 9.70	20.10 ± 2.15 17.91 ± 1.13	0.73 ± 0.08 0.69 ± 0.07	1.37 ± 0.14 1.14 ± 0.06	1.13 ± 0.11 0.89 ± 0.08
OVX (*N* = 5)	EPSCs mEPSCs	4 3	370 314	11.27 ± 8.21 3.07 ± 1.74	17.83 ± 1.15 18.23 ± 1.50	0.64 ± 0.08 0.76 ± 0.17	1.34 ± 0.10 1.34 ± 0.12	1.20 ± 0.10 1.15 ± 0.04
OVX+E (*N* = 5)	EPSCs mEPSCs	8 7	769 496	5.85 ± 7.01 5.85 ± 1.97	22.89 ± 2.29 19.66 ± 1.85	0.63 ± 0.07 0.61 ± 0.06	1.55 ± 0.15 1.50 ± 0.18	1.27 ± 0.12 1.18 ± 0.17
OVX+E+E (*N* = 5)	EPSCs mEPSCs	10 8	1227 1113	15.69 ± 8.40 7.61 ± 3.14	22.57 ± 1.79 21.16 ± 2.01	0.64 ± 0.03 0.65 ± 0.03	1.38 ± 0.09 1.41 ± 0.09	1.19 ± 0.10 1.13 ± 0.13

No statistically significant differences were detected for any parameter between the groups.

**Figure 6.**
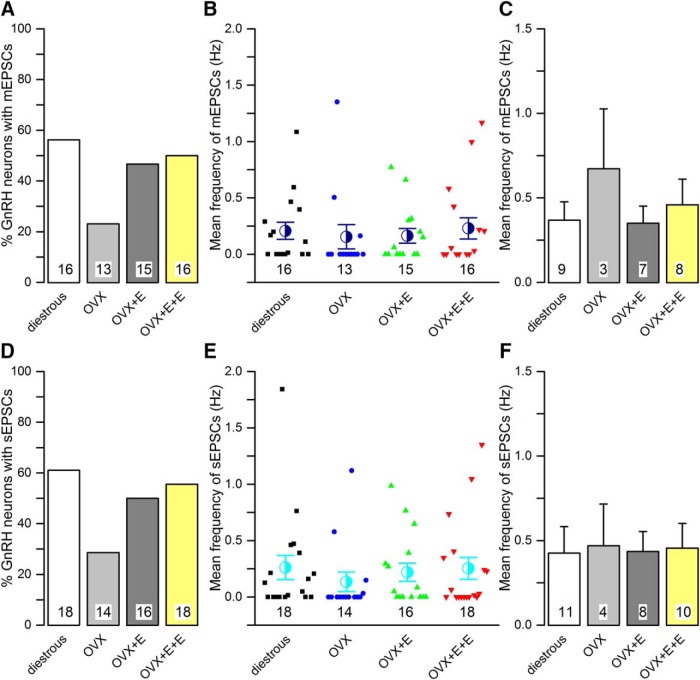
The frequency of excitatory glutamate miniature (mEPSCs) and spontaneous PSCs (sEPSCs) recorded from GnRH neurons do not change in the different mouse models of estrogen negative and positive feedback. ***A***, ***D***, Histograms showing the percentage of GnRH neurons with mEPSCs (***A***) and EPSCs (***D***) in each group. ***B***, ***E***, Scatter plots showing the individual and mean (±SEM) frequency of mEPSCs (***B***) or EPSCs (***E***) in all GnRH neurons of the four experimental groups. ***C***, ***F***, Histograms showing the mean frequency of mEPSCs (***C***) or sEPSCs (***F***) from only cells exhibiting mEPSCs (***C***) or sEPSCs (***F***) in each animal group. Numbers at the base show the cell number. The animal number is five for each group. No statistically significant differences were detected for any parameter between the different groups.

As expected for GnRH neurons (Iremonger et al., 2010), <50% of cells exhibited mEPSCs (Fig. 6*A*). The most noticeable difference was that 56% of GnRH neurons in diestrous mice exhibited mEPSCs compared with only 23% in OVX mice (*p* > 0.05, Fisher’s exact test; Fig. 6*A*). No significant differences were found in the frequency, amplitude or dynamics of mEPSCs between diestrous and OVX mice (Figs. 5, 6*A-C*; Table 2). This was the case regardless of whether we assessed all cells, including those without mEPSCs as a zero value (Fig. 6*B*), or only cells exhibiting mEPSCs (Fig. 6*C*).

No statistically significant differences were found in any mEPSC parameter when comparing OVX, OVX + E, and OVX + E+E animals (Figs. 5, 6*A-C*; Table 2).

### Spontaneous EPSCs (sEPSCs)

**Figure 7.**
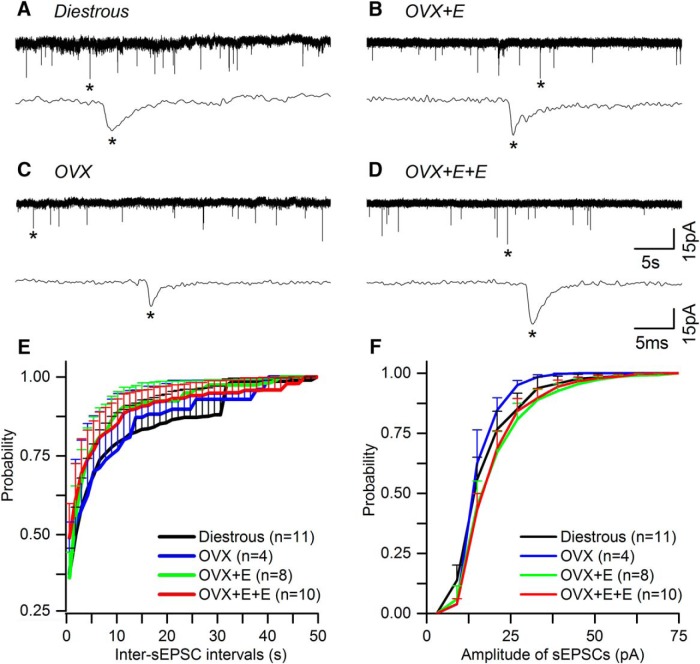
Spontaneous excitatory glutamate receptor PSCs (sEPSCs) recorded from GnRH neurons do not change in the different mouse models of estrogen negative and positive feedback. ***A-D***, Representative examples of sEPSC current recordings in the presence of GABAzine (5 μM) from GnRH neurons in diestrous, OVX, OVX + E, and OVX + E+E mice. Underneath each trace an enlarged time scale of one mEPSC (*) is shown ***E***, ***F***, Cumulative plots of the average inter-sEPSC interval (***E***) and amplitude (***F***) in the different experimental groups. Cell numbers are shown in parenthesis. Numbers of PSCs analyzed is given in Table 2. No statistically significant differences were detected for any parameter between any of the experimental groups.

sEPSCs were recorded from 18 (diestrous), 14 (OVX), 16 (OVX + E), and 18 (OVX + E+E) GnRH neurons, representing 91% of the cells providing mEPSC data above. Recordings undertaken in the presence of GABAzine revealed sEPSCs typical of GnRH neurons (Fig. 7*A-D*).

Sixty percentage of GnRH neurons in diestrous mice exhibited sEPSCs compared with 28% in OVX mice (*p* = 0.08, Fisher’s exact test; Fig. 5*A*). As was found for the mEPSCs, no significant differences were detected for any sEPSC parameter between any of the experimental groups (Figs. 6*D-F*, 7; Table 2).

**Figure 8.**
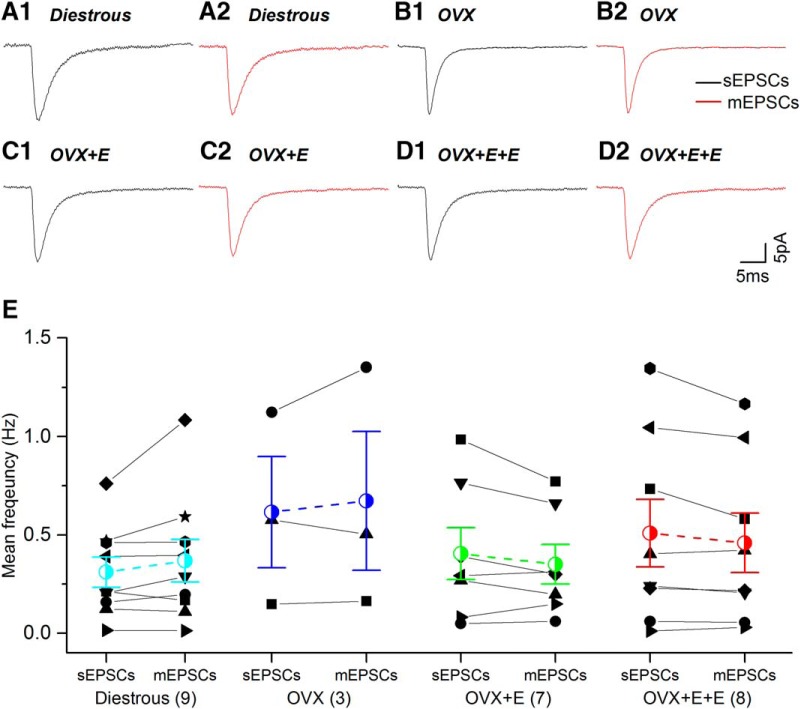
The kinetics and mean frequency of sEPSCs and mEPSCs are not statistically different to one another and do not change during negative and positive feedback. ***A1-D2***, Average waveforms of sEPSCs (black) and mEPSCs (red) are depicted for each experimental group. ***E***, sEPSC and mEPSC values for individual cells (various black symbols) are plotted with group mean (±SEM) indicated in colored symbol with dashed line. Number of individual cells in each group are given in brackets. No statistically significant differences were detected for any parameter between the different groups.

No significant differences were detected between mEPSCs and sEPSCs (Fig. 8; Table 2).

## Discussion

We report here that there are essentially no detectable changes in synaptic ionotropic amino acid receptor dynamics in GnRH neurons in mouse models that mirror physiologic states of estrogen negative and positive feedback. Further, we find no differences in either GABA_A_ or glutamatergic PSCs when comparing diestrous and OVX mice, confirming an absence of change in relation to negative feedback. These observations are in contrast to prior studies using an OVX + E mouse model in which mice exhibit daily LH surges (Christian and Moenter, 2007; Christian et al., 2009). In that model, significant changes in GPSCs and EPSCs were detected in relation to daily switches in negative and positive feedback.

Differences in recording methodology are unlikely to underlie the dissimilarities between our present study and those from the daily surging studies. Recording methods and slice conditions were intentionally very similar between the studies although some technical differences do exist. For example, we use HEPES in our aCSF solution and ATP in our pipette solution, while the studies by Christian and Moenter use a sucrose aCSF cutting solution. These differences would seem unlikely to be responsible for the lack of variation in PSCs in our present studies. Although different transgenic GnRH-GFP mouse lines have been used, this again is unlikely to be significant given that both mouse lines appear to report the great majority of GnRH neurons present in the brain slice (Spergel et al., 1999; Suter et al., 2000). One key observation that was different between studies is the frequency of PSC events detected; in particular GPSCs, where we find GPSCs frequency to be ∼2 Hz compared with ∼0.5 Hz in the Moenter studies (Christian and Moenter, 2007). It remains unclear why we have been able to detect much more frequent GPSCs, and slightly more frequent EPSCs, in our studies. Unfortunately, it is not possible to assess this issue independent of the different OVX + E models used as, to our knowledge, the present report is the first documenting GPSCs and EPSCs in GnRH neurons of intact female mice.

We believe that the primary reason for the discrepancy in GPSC and EPSC findings between studies results from the use of quite different OVX + E mouse models. Under normal circumstances, the LH surge in rodents occurs once every 4-5 d and is driven by a gradual rise in estradiol levels over the preceding 2-3 d coupled to a circadian input that times the surge to beginning of the night (Herbison, 2008; Christian and Moenter, 2010; Levine, 2015). Recent studies undertaken in C57BL6 mice have demonstrated that the proestrous LH surge begins just before lights out and results in peak LH levels of 15-20 ng/ml (Minabe et al., 2011; Czieselsky et al., 2016; Silveira et al., 2017). The OVX + E+E model used in the present study was designed by Bronson following a meticulous series of experiments using different estradiol replacements regimens aimed at mirroring the rising follicular phase levels of circulating estradiol (Bronson, 1981). This protocol results in an LH surge beginning just before lights out but with peak LH levels of ∼8 ng/ml, being approximately half that found in proestrous mice (Czieselsky et al., 2016). The OVX + E model used by the Moenter laboratory is intentionally designed to maintain constant high proestrous levels of estradiol so as to induce both negative and positive feedback every day for several days (Christian et al., 2005). This model generates a daily LH surge at the time of lights out although the amplitude of these surges is very low at ∼1 ng/ml; 10- to 15-fold less than that of a proestrous surge (Christian et al., 2005; Silveira et al., 2017). Thus, there are profound differences between the mouse models used for PSCs analysis in GnRH neurons; one exhibits daily transitions from negative to positive feedback that are driven by circadian inputs and independent of circulating estradiol concentrations, while the other examines negative and positive feedback in a model of the natural estrous cycle.

We demonstrate that no substantial changes occur in the frequency of GABA_A_ or glutamate PSCs in GnRH neurons in relation to negative or positive feedback. This result is perhaps surprising given the substantial data implicating roles of GABA and glutamate in regulating GnRH neurons at times of estrogen negative and positive feedback. For example, using cell-specific deletion of *Esr1* (estrogen receptor-α), it was recently reported that estrogen-dependent GABA signaling was required for normal positive feedback whereas estrogen-dependent glutamatergic transmission was necessary for both negative and positive feedback (Cheong et al., 2015). Also, the numbers of dendritic spines detected on the population of GnRH neurons involved in the generating the GnRH surge increases in OVX + E+E mice at the time of the LH surge (Chan et al., 2011).

However, it is important to recognize a number of caveats when using the brain slice approach. First, the great majority of PSCs recorded in GnRH neurons do not result from action potential-dependent GABA or glutamate release. As found in all such studies (Sim et al., 2000; Christian and Moenter, 2007; Christian et al., 2009; Penatti et al., 2010), the addition of TTX to the perfusion medium makes no substantial difference to the frequency of PSCs. Second, although the standard approach, GPSCs and EPSCs are measured using a whole-cell patch that results in the contents of the cell being dialyzed with that of the pipette electrode. The effects of this on PSCs amplitude, in particular, are unclear. Third, we note that neurons within the GnRH neuronal network will experience a fall in extracellular estradiol concentrations on removal from the brain and this is not supplemented in the bathing aCSF. Prior brain slice studies have shown significant effects from the sudden application of high estradiol concentrations on GnRH neuron GPSC frequency (Romanò et al., 2008; Chu et al., 2009). These and other factors may be of significance when interpreting the results of brain slice studies. Certainly, it is clear that the patterns of GnRH neuron firing *in vivo* are different to what is found in the brain slice (Constantin et al., 2013). Thus, it remains possible that substantial changes in GABA and glutamatergic drive to the GnRH neuron exist across the estrous cycle but, at present, it is not possible to examine these changes *in vivo*.

The kinetics of GPSCs and EPSCs in GnRH neurons were not altered in any of the OVX ± E or diestrous groups. This suggests that no substantial changes are occurring in the subunit composition and/or post-translational modification of GABA_A_, AMPA and NMDA receptors expressed on the cell bodies and proximal dendrite of GnRH neurons. This is compatible with the absence of the key estrogen receptor ESR1 in GnRH neurons. Prior work indicates that the broad range of GABA_A_ receptor subunits expressed by GnRH neuron narrows across postnatal development (Sim et al., 2000) but, nevertheless, many different GABA_A_ receptor subunits remain expressed by adult GnRH neurons (Pape et al., 2001; Todman et al., 2005; Penatti et al., 2010; Vastagh et al., 2016). Seemingly, these subunits are capable of generating multiple different GABA_A_ receptors that, nevertheless, are shown here to remain stable during negative and positive feedback. The same situation exists for glutamate EPSCs that, in our recordings, represent primarily AMPA PSCs. However, we note findings in the rat where a re-arrangement of AMPA receptor subunit stoichiometry within GnRH neurons is thought to be responsible for an increase in AMPA mEPSCs frequency on proestrus (Tada et al., 2013).

In summary, we report here that ionotropic GABA and glutamate signaling to the GnRH neuron is not substantially altered in OVX + E mice that model the normal estrogen negative and positive feedback processes. This does not discount the involvement or importance of amino acid signaling in these processes but does suggest that ionotropic amino acid receptor expression, at least, remains relatively stable. The observations also highlight the substantial differences that can exist between different OVX + E mouse models. The OVX + E daily surging models are not designed to mimic the normal stages of the estrous cycle but do, nevertheless, demonstrate the powerful impact of circadian influences on the GnRH neuron (Christian and Moenter, 2010). In this light, it is interesting to observe that the changes in GPCSs and EPSCs encountered in that model are not found in the present model with fluctuating estrogen levels. Over and above these issues, however, these studies reinforce the need for a unified model of estrogen negative and positive feedback that can be used across laboratories examining the neurobiology of the GnRH neuron.

## Synthesis

Reviewing Editor: Margaret McCarthy, Univ of Maryland School of Medicine Decisions are customarily a result of the Reviewing Editor and the peer reviewers coming together and discussing their recommendations until a consensus is reached. When revisions are invited, a fact-based synthesis statement explaining their decision and outlining what is needed to prepare a revision will be listed below. The following reviewer(s) agreed to reveal their identity: Istvan Abraham, Dai Mitsushima

In this provocative work, authors re-examined the role of positive and negative E2 feedback on GABA and glutamate receptor signaling in GnRH neurons using whole cell patch clamp electrophysiology. They applied a well-characterized E2 feedback model that is one of best in the estrous cycle model ‘market’. In contrast to previous studies, they found no difference in GABAA-PSCs or glutamate-PSCs during the time of negative or positive feedback. While this work would be an important step for elucidating the mechanism of estrogen feedback action on GnRH neurons it is suffers form the typical problems of publishing negative results. The authors are in a difficult position because publishing negative results requires more control experiments than usual and plenty of considerations.

Both reviewers felt there needed to be a greater emphasis on the measurement of minis as opposed to exclusively relying on PSCs. The reviewers argue that:

1) Concerted understanding of the impact of E2 feedback on electrical activity of GnRH neurons requires information on both sPSC and mPSC. I think mPSCs are also important because it describes single vesicle release and E2 is known to modulate this. In addition, previous experiments describe that E2 initiates burst of mISPC in GnRh neurons (Romano et al., Endocrinology, 2008, 149:5335).

2) Basically, it is difficult to interpret the results of the PSCs. Instead of PSCs, the authors should use mGPSCs and mEPSCs as the main results of the manuscript. The results of mGPSC or mEPSC indicate the postsynaptic currents induced by a single vesicle of GABA or glutamate. According to the Tables, the authors seem to have many mini data already, although they may need to add some data. There is no reason to throw away the spontaneous PSC data. However, I believe that main data have to be mini, while the spontaneous PSC data is additional supporting data.

The reviewers had several other suggestions for improvement as well:

1) The whole cell patch clamp is one of best techniques for detecting PSCs. However, in whole cell studies dilution of cytosol by the pipette solution may cause ion channel dysfunction that may interfere with the actual E2-induced action and/or GABA or glutamate receptor electrophysiology. The authors may want to discuss this. There are several minimizing method exist to compensate the dilution effect (e.g. IEEE Trans Biomed Eng., 2013 ;60:2042-51). The authors can apply one of these techniques to repeat the critical part of the experiments.

2) The authors pointed out that the E2 feedback story is based upon the expression of estrogen receptors in GnRH neurons. The in vivo experiments highlighted the importance the classical estrogen receptors in feedback mechanisms. However the acute brain slice is in hypoxic condition and it is very likely that the expression of the GPER1 increased in the hypothalamus (review see AAPS J. 2016, 18: 305-310). Accordingly the ER/GPER1 ratio changed and E2 receptor signaling mechanisms can be altered in the GnRH neurons of acute brain slices. The author may want to repeat the key points of the experiment in hypoxic-free condition using higher O2 concentration and/or a different incubation chamber.

3) Previous work showed the acute 100nM E2 treatment induced burst of mIPSC in GnRH neurons (Endocrinology 2008, 149:5335-5344). Although I am highly aware that the E2 concentration was pharmacological these results are somewhat contradictory and it raises the possibility of control study. If the E2 concentration is around 100 pM in the brain at the time of the estrus it would be interesting to examine the effect of 100 pM of E2 on GPSCs and EPSCs on acute brain slice from ‘OVX+E mice’. This experiment can serve as control for E2 feedback action for brain slices recorded at ‘surge onset’.

4) Figure 1 and 4 representative traces would be improved if the authors used enlarged traces on the time axis to distinguish the responses from noise.

5) The authors appear to have recorded the PSCs with the exposure to excitation light? Exposure to the excitation light should be avoided during the recordings, though the light is necessary to specify GnRH neurons. Can the authors speak to this issue?
